# COVID‐19‐associated secondary hemophagocytic lymphohistiocytosis requiring hematopoietic cell transplant

**DOI:** 10.1002/jha2.456

**Published:** 2022-05-11

**Authors:** Jacob R. Greenmyer, Kirk D Wyatt, Sam Milanovich, Mira A. Kohorst, Asmaa Ferdjallah

**Affiliations:** ^1^ Pediatric and Adolescent Medicine Mayo Clinic Rochester Minnesota USA; ^2^ Pediatric Hematology and Oncology Sanford Health Fargo North Dakota USA; ^3^ Pediatric Hematology and Oncology Mayo Clinic Rochester Minnesota USA

**Keywords:** hematology, infection, pediatrics, stem cell transplantation, thrombocytopenia

## Abstract

Coronavirus disease 2019 (COVID‐19) infection causes a variety of extrapulmonary complications in pediatric patients. Multisystem inflammatory syndrome and hemophagocytic lymphohistiocytosis (HLH) are related to hypercytokinemia in COVID‐19 patients. HLH is a disorder of exaggerated inflammation resulting in a cytokine storm and unrestricted hemophagocytosis. HLH can be primary (familial) or secondary (acquired). Secondary HLH (sHLH) can occur in patients with rheumatologic, oncologic, or infectious diseases. The link between COVID‐19 and HLH has been reported in pediatric patients. Here we report a case of a pediatric patient who developed refractory sHLH secondary to COVID‐19 infection and required a hematopoietic cell transplant for the cure.

## 1 INTRODUCTION

Coronavirus disease 2019 (COVID‐19) infection causes a variety of extrapulmonary complications in pediatric patients including multisystem inflammatory syndrome (MIS‐C) and hemophagocytic lymphohistiocytosis (HLH) [1, 2]. Complications such as MIS‐C and HLH are related to hypercytokinemia in COVID‐19 patients [2, 3, 4].

HLH is a hematologic disorder of exaggerated inflammation that results in a sepsis‐like cytokine storm and unrestricted hemophagocytosis [5]. HLH can be primary (familial) or secondary (acquired). Secondary HLH (sHLH) can occur in patients with rheumatologic, oncologic, or infectious diseases [5]. A case of COVID‐19 inducing HLH has previously been reported in a 6‐week‐old infant with Chediak‐Higashi syndrome [6]. Here we report a case of a pediatric patient who developed refractory sHLH to COVID‐19 infection and required a hematopoietic cell transplant (HCT) for the cure.

## 2 CASE

A 5‐year‐old female presented to her pediatrician with fevers (101–105°F) and papular rash for three days and was subsequently diagnosed with polymerase chain reaction (PCR) positive COVID‐19. A chest X‐ray was unremarkable, and she was treated empirically with cefdinir and azithromycin. After 4.5 weeks of persistent fevers despite antibiotic therapy, she was transferred to a tertiary center for further evaluation with concern for further evaluation of her hyperinflammatory state.

Labs at the time of re‐evaluation are summarized in Table 1. A repeat COVID‐19 PCR test was negative, indicating clearance of the infectious agent. The differential diagnosis for the hyperinflammatory state in this patient included systemic infection, MIS‐C, and HLH. It was possible that the finding of a positive COVID‐19 PCR was coincidental; therefore, we considered a systemic infection or post‐infection hyperinflammatory state caused by an etiology other than COVID‐19. Blood and urine cultures obtained were negative. Histoplasma, Blastomyces, HIV, and Monospot testing were also negative. Other than COVID‐19 PCR positivity, there was no infectious etiology identified. Although the identification of severe acute respiratory syndrome coronavirus 2 (SARS‐CoV‐2) is temporally related with the hyperinflammation, it is possible that other unfound causes may have been at play.

**TABLE 1:** Laboratory results at the time of re‐evaluation

**Lab**	**Value**	**Reference range**
Hemoglobin	8.9 g/dl	10.5–14.5 g/dl
Mean corpuscular volume	76.5 fl	74.0–89.0 fl
Platelets	157,000/ul	140,000–400,000/ul
White blood cells	36,200/ul	5,000–15,000/ul
Alkaline phosphatase	559 U/L	40–150 U/L
Alanine aminotransferase	312 U/L	6–55 U/L
Aspartate aminotransferase	767 U/L	5–34 U/L
C‐reactive protein	178.8 mg/L	0.0–8.0 mg/L
Erythrocyte sedimentation rate	44 mm/h	3–13 mm/h
Procalcitonin	9.59 ng/ml	<0.07 ng/ml
Ferritin	>40,000 ng/ml	15–80 ng/ml
Triglyceride	673 mg/dl	50–150 mg/dl
Fibrinogen	310 mg/dl	200–450 mg/dl

Strong consideration was given to MIS‐C, which was the leading differential diagnosis at repeat evaluation. The diagnosis of MIS‐C requires (1) fever, inflammation, and multisystem (>2) involvement, (2) current or recent SARS‐CoV‐2 infection or exposure, and (3) no alternative plausible diagnosis [7]. This patient had (1) fever, inflammation, and multisystem involvement (dermatologic, hematologic) and (2) positive SARS‐CoV‐2 infection. However, there were several features of this patient's clinical presentation that prompted us to consider alternative diagnoses. First, while any two systems can be involved in MIS‐C, patients with MIS‐C often have clinical gastrointestinal and cardiac symptoms (82% and 66%, respectively) [8]. Gastrointestinal symptoms are often nausea, vomiting, abdominal pain, and/or diarrhea, all of which were absent in this patient. Second, patients are frequently (76% of the time) clinically unstable with rapidly and progressive symptoms that require intensive support [8]. Third, while anemia classifies as a hematologic manifestation, lymphocytopenia and thrombocytopenia are more characteristic of MIS‐C [9]. Fourth, while ferritin is often elevated in severe, MIS‐C, the elevation of ferritin is frequently much more modest [10]. In a study that evaluated features of MIS‐C with cytokine storm syndrome scoring systems, the highest reported ferritin level was 1527.6 ng/ml [10]. We considered HLH as an alternative diagnosis based on absence of clinical gastrointestinal and cardiac symptoms, prolonged hemodynamic stability, absence of leukopenia, and profound hyperferritinemia.

**FIGURE 1:**
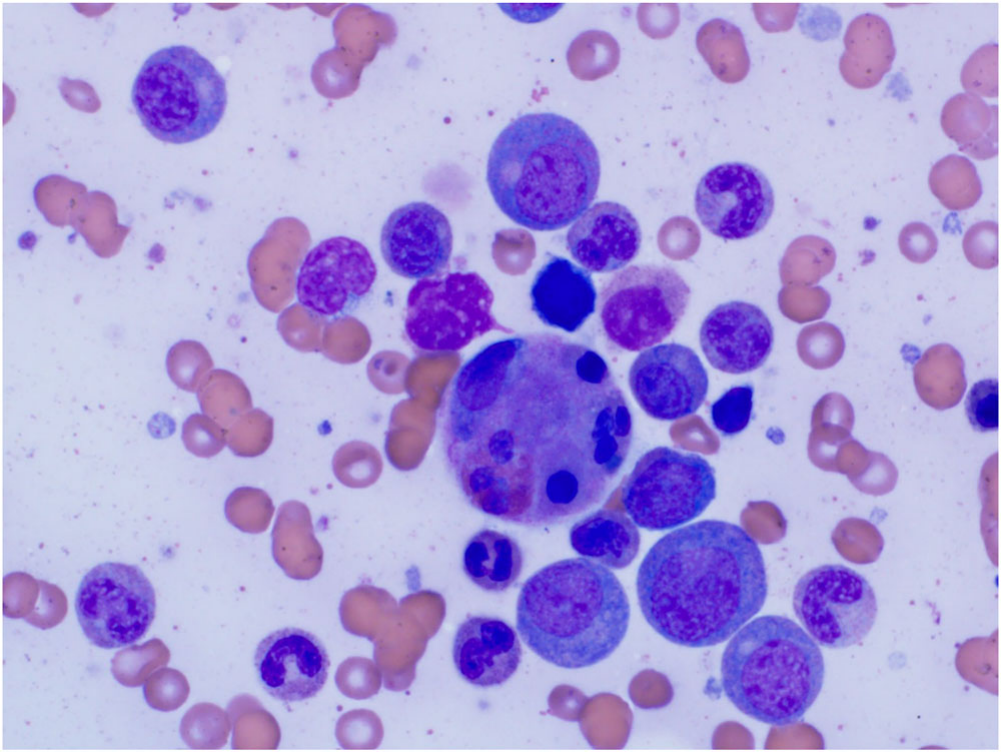
A bone marrow biopsy and aspirate demonstrating normal cellularity with rare hemophagocytosis

Further workup for HLH revealed mildly decreased natural killer (NK) cell function (NK lytic units 0.9, normal >/= 1.0; via chromium release methodology) and an elevated soluble interleukin‐2 receptor (sIL‐2R) (3690 U/ml: reference range 144–1329 U/ml). Fasting triglycerides were 673 mg/dl (nl 50–150 mg/dl). A bone marrow biopsy and aspirate demonstrated normal cellularity with rare hemophagocytosis (Figure 1). No monoclonal population was identified on flow cytometry. Immunoglobin (IgA, IgG, and IgM) testing was normal. Quantitative lymphocyte subsets revealed mildly low %CD4 T cells (19%, nl 30%–56%), low absolute CD4 T cells (266, nl 497–2267 cells/mcl), and elevated % CD8 T cells (41%, nl 16%–34%).

Cerebrospinal fluid (CSF) analysis demonstrated pleocytosis and an elevated white blood cell count of 57/ul (reference range 0–10/ul) in the setting of an atraumatic lumbar puncture and normal CSF protein (43.0 mg/dl: reference range 15.0–45.0 mg/dl). CSF cytology revealed a mixture of leukocytes without hemophagocytosis. CSF culture was negative.

A CT scan of the chest, abdomen, and pelvis demonstrated hepatosplenomegaly, gallbladder wall thickening, small volume ascites, and multiple areas of mild lymphadenopathy. An echocardiogram and EKG were normal.

The diagnostic criteria for HLH include fever, splenomegaly, hypertriglyceridemia, elevated ferritin, hemophagocytosis, decreased NK cell activity, and elevated sIL‐2R (Table 2) [11, 12]. This case met 7/8 diagnostic criteria for HLH with only 5/8 being necessary for diagnosis (Table 2). H‐Score was 236, which portends a 98%–99% probability of HLH [13]. Of note, in the cohort reported by Reiff and Cron, the median H‐Score in patients with MIS‐C was 68 with an interquartile range of 49–79 and there were zero patients with MIS‐C that had an H‐Score > 169 [10].

**TABLE 2:** Criteria of hemophagocytic lymphohistiocytosis (HLH) and H‐Score

**Clinical and Laboratory Criteria**			
Clinical criteria	Demonstrated by patient	H‐Score criteria	H‐Score points for patient
Fever	Yes	Fever	49 points (*T*_max_ 40.5°C)
Splenomegaly	Yes	Organomegaly	38 points (hepatomegaly and splenomegaly)
Cytopenias (2 or 3 lineages) ‐ Hemoglobin (<100 g/L in infants <4 weeks) ‐ Platelets < 100 × 10^9^/L ‐ Neutrophils < 1.0 × 10^9^/L	No Yes No No	Cytopenias	0 points (1 lineage)
Ferritin ≥ 500 mg/L	Yes	Hyperferritinemia	50 points (> 6000 ng/ml)
Hemophagocytosis	Yes	Hemophagocytosis in bone marrow?	35 points (Yes)
Triglycerides ‐ Fasting triglycerides ≥ 3.0 mmol/L ‐ Fibrinogen ≤ 1.5 g/L	Yes Yes No	Triglycerides	64 points (> 4 mmol/L)
Low/absent NK‐cell activity	Yes	N/A	
Elevated soluble CD25/IL‐2 receptor (≥ 2400 U/ml)	Yes	N/A	
Serum glutamic oxaloacetic transaminase (SGOT)	N/A	SGOT	0 points (Not drawn in this patient)
Total diagnostic criteria (5 of 8 required)	Yes (7 of 8)	H‐Score total	236*
**Genetic Criteria**			
Gene Panel		No	

* 98%–99% probability of hemophagocytic syndrome according to H‐Score [13].

Based on our diagnosis of HLH, a comprehensive inherited HLH genetic panel of 21 genes associated with familial HLH was negative for pathogenic variants (Table S1) [11, 12]. Therefore, the patient was considered to have acquired HLH secondary to COVID‐19. By contrast, the case described by Lange was a case of fHLH (Chediak‐Higashi syndrome) triggered by COVID‐19 [6].

Treatment was started per‐protocol HLH‐94 with etoposide 150 mg/m^2^ twice weekly and dexamethasone 5 mg/m^2^ twice daily [14]. Intrathecal methotrexate was not initiated given lack of CNS involvement. Within 2 days, her fevers resolved, laboratory parameters improved, and she was discharged home.

Per protocol, dexamethasone and etoposide were tapered; dexamethasone from 10 to 2.5 mg/m^2^/day and etoposide from twice weekly dosing to weekly. After the taper she developed fevers again. Her cell counts at that time included a white blood cell of 21,800/ul with a neutrophilic predominance and decrease in hemoglobin to 7.9 mg/dl. The ferritin increased to 34,832 ng/ml and sIL‐2R was elevated at 4310 U/ml. Dexamethasone was increased to 5 mg/m^2^ twice daily and plans were made to proceed to HCT.

Upon dexamethasone escalation, she experienced a rapid clinical response with resolution of fevers and improved ferritin. Dexamethasone was then gradually weaned to avoid relapse of her sHLH. As a consequence of prolonged dexamethasone use, she developed myopathy, hyperglycemia requiring insulin, thrush, and significant weight gain with Cushingoid features. Emapalumab was initiated as a steroid‐sparing agent, at a dose of 1 mg/kg twice weekly, etoposide was discontinued, and corticosteroids were tapered to physiologic dosing [15]. She tolerated steroid taper without recurrence of active HLH. Prior to transplant, her ferritin, NK cell function, and sIL‐2R normalized.

She underwent reduced‐intensity conditioning with fludarabine (cumulative dose 160 mg/m^2^), melphalan (cumulative dose 140 mg/m^2^), and TBI (2 Gy) followed by a paternal haploidentical allogeneic HCT. Her graft‐versus‐host‐disease prophylaxis consisted of post‐transplant cyclophosphamide (50 mg/kg on day +3 and day +4), tacrolimus and mycophenolate mofetil. Day +30, +70, and +100 peripheral blood chimerism demonstrated 100% donor (in both CD3 and CD33 compartments). Her transplant course was complicated by engraftment syndrome requiring high‐dose steroids; respiratory failure requiring intubation and mechanical ventilation; hyperglycemia and hypertriglyceridemia; secondary adrenal insufficiency; veno‐occlusive disease requiring defibrotide; and transplant‐associated thrombotic microangiopathy requiring eculizumab. She is now 8 months status‐post transplant, remains in remission, is transfusion independent.

## 3 DISCUSSION

HLH is a disorder of uninhibited inflammation and hemophagocytosis that can be secondary to infection such as COVID‐19. To our knowledge, this is the first case of a pediatric patient with COVID‐19 associated sHLH successfully treated with Emapalumab followed by HCT. Hence, similarly to other infections, COVID‐19 can cause refractory sHLH unresponsive to standard therapy, ultimately requiring HCT in pediatric patients. This report highlights the potential severity of COVID‐19 in pediatric patients, despite its otherwise typical mild course.

## 4 LIMITATIONS

While we excluded numerous causes of hyperinflammation and identified SARS‐CoV‐2 as an etiology in this case, we cannot definitively exclude the possibility of other contributors to hyperinflammation in this patient.

## CONFLICT OF INTEREST

The authors declare they have no conflicts of interest.

## Supporting information



Supporting information
