# Supplementary material for: COVID‐19‐associated secondary hemophagocytic lymphohistiocytosis requiring hematopoietic cell transplant
Source: EJHaem. 2022 May 11;3(3):1025–8. doi: 10.1002/jha2.456 (PMC9348183; doi:10.1002/jha2.456)
Supplement: Supplementary file 1 — Supporting information [file JHA2-3-1025-s001.docx]

| **Variants identified in the HLH Panel from *Invitae Genetics Laboratories*** |
| --- |
| ADA, AP3B1, BLOC1S6, BTK, CD27, IL2RA, IL2RG, ITK, LYST, MAGT1, MVK, PNP, PRF1, RAB27A, SH2D1A, SLC7A7, STX11, STXBP2, UNC13D, WAS, XIAP |
| If the initial panel is negative, reflex to: AP3D1, CTPS1, GATA2, NLRC4. |

Supplemental table 1: Variants identified in the HLH Panel from *Invitae Genetics Laboratories*
